# Basic Taste Stimuli Elicit Unique Responses in Facial Skin Blood Flow

**DOI:** 10.1371/journal.pone.0028236

**Published:** 2011-12-01

**Authors:** Hideaki Kashima, Naoyuki Hayashi

**Affiliations:** 1 Graduate School of Human-Environment Studies, Kyushu University, Fukuoka, Japan; 2 Institute of Health Science, Kyushu University, Fukuoka, Japan; Hungarian Academy of Sciences, Hungary

## Abstract

Facial expression changes characteristically with the emotions induced by basic tastes in humans. We tested the hypothesis that the five basic tastes also elicit unique responses in facial skin blood flow. Facial skin blood flow was measured using laser speckle flowgraphy in 16 healthy subjects before and during the application of basic taste stimuli in the oral cavity for 20 s. The skin blood flow in the eyelid increased in response to sweet and umami taste stimuli, while that in the nose decreased in response to a bitter stimulus. There was a significant correlation between the subjective hedonic scores accompanying these taste stimuli and the above changes in skin blood flow. These results demonstrate that sweet, umami, and bitter tastes induce unique changes in facial skin blood flow that reflect subjective hedonic scores.

## Introduction

The gustatory characteristics of foods exert effects on eating behaviors and judgments of their acceptability so as to avoid the ingestion of toxic and unpleasant foods [1]. One way of conferring these characteristics to one's peers is by facial expressions, since those associated with the intake of foods with pleasant and unpleasant tastes are consistent among different cultures, races, and individuals [2]. This has led to facial expression being used to evaluate objective gustatory senses [3]. However, facial expressions are also generated by voluntary muscles via the motor nervous system, and can easily be modulated or masked by one's intention. This has led to a lack of objectivity regarding the evaluation of facial expression in response to taste.

Contrary to the responses induced by voluntary muscles, it is difficult to intentionally modulate or mask a response that is induced by the autonomic nervous system (ANS), since it is not under voluntary control. ANS activity is affected by hedonic valence and emotion, and so responses identified by the ANS are frequently used as lie detectors. Some previous studies have suggested that the basic tastes also induce specific heart rate and finger skin resistance responses according to the associated hedonic valence or emotion [4]–[6]. These studies implicated the existence of unique ANS responses to hedonic valence or emotion. Our previous study also suggested that gustatory information increases the blood flow in the gastrointestinal tract [7]. However, such responses cannot be used as a tool by which to communicate with peers since most of them occur inside the body (i.e., playing a functional role in digestion). It is unknown whether ANS responses such as changes in facial skin blood flow mirror gustatory information.

The facial skin blood flow responses are induced by the ANS [8], [9] and can be visible to others [10]. The blood flow response is involuntary and rapid, and so it is readily relayed to peers. We therefore hypothesized that facial skin blood flow reflects our emotion related to the tastes we are experiencing. To test this hypothesis, we observed the facial skin blood flow responses to five basic tastes applied in three different concentrations to the oral cavity. We found that pleasant and unpleasant tastes elicit unique changes in facial skin blood flow that reflect subjective hedonic scores.

## Results

**Figure 1:**
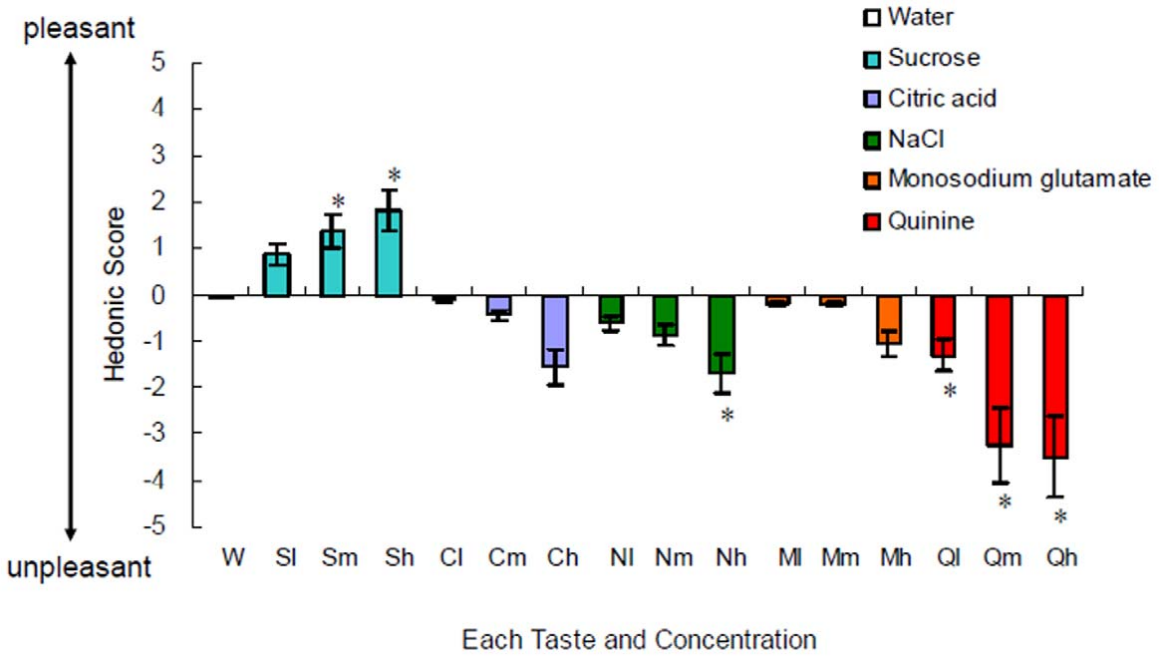
Hedonic scores (mean and SEM values) related to each taste, from −5 = (the most unpleasant) to +5 ( = the most pleasant). Each taste was compared to water by multiple comparisons (Bonferroni's post-hoc test). *P<0.05. W, water; S, sweet; C, citric acid; N, NaCl; M, monosodium glutamate; Q, quinine; l, low concentration; m, medium concentration; h, high concentration.

The taste stimuli successfully provoked various hedonic valences, with the hedonic scores to basic tastes differing significantly from that to the water control (Fig. 1). The pleasant score was higher for medium and high concentrations of sucrose (sweet) stimuli than for the water control [repeated-measures analysis of variance (ANOVA): F_1, 15_ = 12.89, P<0.05]. The unpleasant score was higher for a high concentration NaCl (salty) stimulus and all concentrations of quinine (bitter) than for the water (repeated-measures ANOVA: salty, F_1, 15_ = 13.41, P<0.05; bitter, F_1, 15_ = 57.88, P<0.05). Citric acid (sour) and monosodium glutamate (umami) stimuli were rated as neither pleasant nor unpleasant compared to the water (repeated-measures ANOVA: sour, F_1, 15_ = 3.05, P>0.05; umami, F_1, 15_ = 2.09, P>0.05).

**Figure 2:**
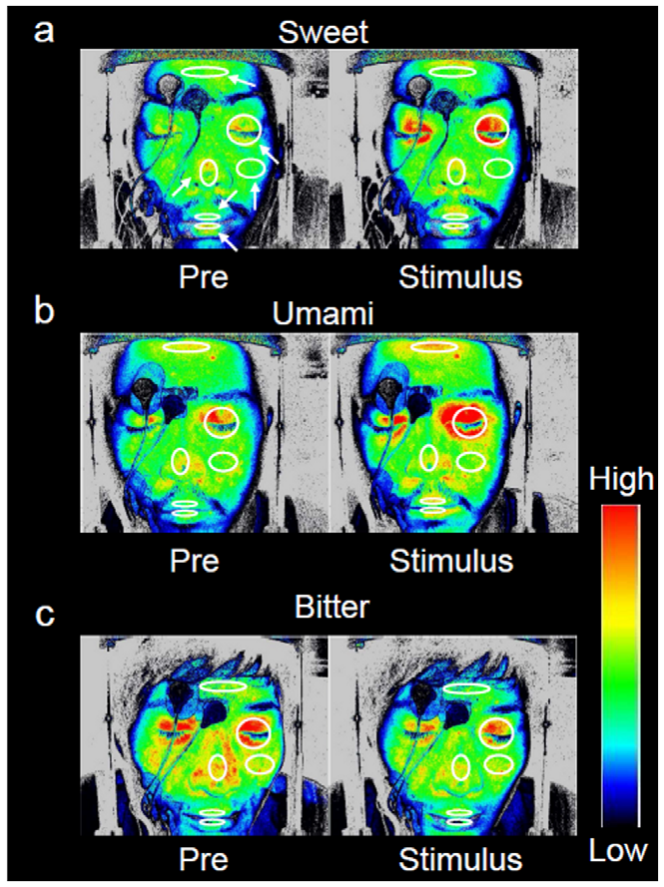
Changes in facial skin blood-flow distribution in a subject before (Pre) and during (Stimulus) gustatory stimulation. The colored bar on the right side indicates the blood flow magnitude, with the red and blue colors indicating higher and lower blood flows, respectively. The white ellipses in the six facial regions represent the target areas where blood flow was measured. Sweet (a) and umami (b) stimuli characteristically increased eyelid skin blood flow, while bitter (c) stimuli decreased nose skin blood flow.

**Figure 3:**
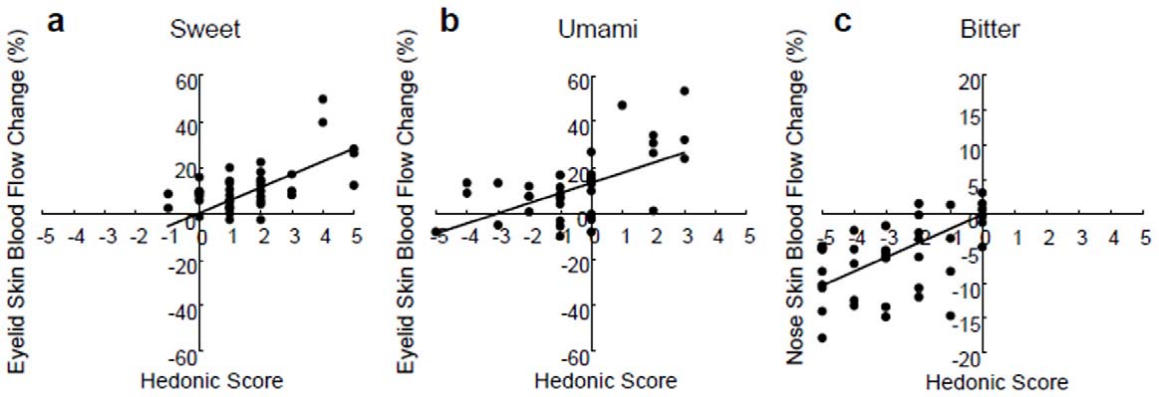
Sweet, umami, and bitter stimuli were significantly correlated with hedonic rating and blood flow in specific facial skin regions. Sweet (a) and umami (b) stimuli were significantly correlated with hedonic rating and eyelid skin blood flow (sweet: r = 0.60, n = 41; umami: r = 0.57, n = 44). Bitter (c) stimuli were significantly correlated with hedonic rating and nose skin blood flow (r = 0.52, n = 38).

Sweet and umami stimuli were reflected in the eyelid skin blood flow, such that the blood flow increased with the hedonic score (Fig. 2a, b). We computed the relative change in facial skin blood flow induced by each taste stimulus as compared to the baseline before the stimulus. Table 1 indicates the relative changes in skin blood flow to each taste stimulus. The eyelid skin blood flow significantly increased for all concentrations of sweet and umami stimuli. The hedonic score was significantly and positively correlated with changes in eyelid skin blood flow for sweet (Fig. 3a; r = 0.60, F_1, 40_ = 22.62, P<0.05) and umami (Fig. 3b; r = 0.57, F_1, 43_ = 20.35, P<0.05) stimuli. The slopes and intercepts were similar for both relationships. Medium and high concentrations of bitter stimuli were also reflected in the nose skin blood flow, which decreased in all subjects (Fig. 2c), and were significantly correlated with the hedonic score (Fig. 3c; r = 0.52, F_1, 37_ = 13.90, P<0.05).

**Table 1:** Changes in mean facial skin blood flow in response to various taste stimuli.

	Taste concentration	Forehead	Eyelid	Nose	Cheek	Upper lip	Lower lip
Water		1±2*	5±2*	0±1*	4±2*	42±5*	10±3*
Sweet	Low	−4±2*	8±2*	−1±2*	2±2*	46±7*	8±3*
	Medium	−7±2*	13±4*	−3±1*	−1±1*	38±7*	13±6*
	High	−4±1*	12±3*	−1±2*	−1±2*	43±8*	13±5*
Sour	Low	1±2*	14±3*	1±2*	3±2*	47±5*	9±4*
	Medium	0±2*	11±3*	3±2*	7±2*	55±4*	18±7*
	High	−2±2*	14±3*	1±3*	8±2*	55±9*	13±5*
Salty	Low	−2±2*	5±4*	−3±2*	1±3*	51±5*	12±6*
	Medium	−1±2*	11±3*	1±3*	4±2*	42±4*	14±5*
	High	−5±2*	6±3*	1±2*	5±2*	46±5*	17±5*
Umami	Low	0±2*	10±3*	1±1*	3±2*	50±7*	8±5*
	Medium	0±2*	10±3*	−1±2*	1±1*	46±6*	14±5*
	High	−1±2*	13±5*	1±2*	2±2*	47±6*	16±4*
Bitter	Low	−3±2*	2±3*	−3±1*	−3±2*	37±5*	10±5*
	Medium	−2±2*	1±3*	−9±2*	−2±1*	42±5*	14±6*
	High	0±1*	5±3*	−6±1*	2±2*	44±5*	25±7*

Data are mean and SEM values. n = 16.

*P<0.05 vs. baseline.

While responses in facial skin blood flow were observed in other facial regions (Table 1), their magnitudes were not related to hedonic valence. Forehead skin blood flow significantly decreased in response to all concentrations of sweet and high concentration salty stimuli. Eyelid skin blood flow significantly increased in response to all concentrations of sour and medium concentrations of salty stimuli. Cheek skin blood flow significantly increased in response to medium and high concentrations of sour and high concentration of salty stimuli. Upper lip blood flow significantly increased in response to all stimuli, whereas lower lip blood flow significantly increased only in response to taste stimuli at medium and high concentrations. The changes in skin blood flow in each region were not linearly related to the solution concentration.

The heart rate, mean arterial blood pressure (MAP), and electromyographic activities of the zygomaticus major and corrugator supercilii muscles did not significantly change from baseline in response to any of the taste stimuli.

## Discussion

The present study demonstrated that basic tastes elicit unique responses in facial skin blood flow, and that the magnitude of these responses is related to hedonic valence but not simply to the solution concentration. Facial skin blood flow is altered by the activity of the ANS [8], [9]. The findings of the present study support the hypothesis that gustatory information is reflected in characteristic facial skin blood flow responses induced by the ANS.

Putting any substance into the oral cavity can elicit a blood flow response in the upper lip, since even pure water increased skin blood flow in this area. However, the responses elicited in other regions must have been due to the actual taste stimuli.

The unpleasant rating for bitter stimulus was associated with changes in nose skin blood flow. Unpleasant stimuli, such as bitterness, are important cues to remind us of the danger of toxic foods, making us experience a related negative emotion [1]. Many species including monkeys, cats, and human infants exhibit distaste in response to bitterness [11]. Thus, a reduction in nose skin blood flow in response to a bitter stimulus may help to express the hazard related to certain foods in humans as well as other species.

The pleasant rating of sweet and umami stimuli was associated with an increase in eyelid skin blood flow. This increase is an important cue to remind us of the essential macronutrients that provide the energy we need to survive. The increase in eyelid skin blood flow in response to sweet and umami stimuli may help to inform peers of the utility of this food. The eyelid or face could reflect the hedonic valence better than other areas of the body. A previous study found that sweet stimuli associated with different hedonic scores and taste intensities were not related to changes in vascular resistance in finger skin [12]. The differences between the results of the present and previous studies may be attributable to the different target areas studied—it has been shown previously that facial skin area reflects emotion more effectively than does finger skin [13].

The umami stimulus did not significantly change the hedonic score in the present study, which is consistent with previous reports [14]. The umami taste is not pleasant for all subjects. Nevertheless, the hedonic rating in response to the umami stimulus was significantly correlated with the change in eyelid skin blood flow, since hedonic valence was associated with the change in blood flow during both the umami and sweet stimuli. This shows that the umami stimulus itself was not necessarily responsible for the increase in blood flow in the eyelid.

Sweet and umami stimuli activate similar cortical regions in the brain [15]. The hedonic valence to umami stimuli varies widely among individuals, and those who perceived the umami stimulus as a pleasant taste exhibited an increase in blood flow in the eyelid. Eyelid skin blood flow must be affected by the hedonic score in response to sweet and umami stimuli.

The mechanism by which bitter stimuli decrease the skin blood flow in the nose area may be explained by anatomical and physiological characteristics. The nose area has many arteriovenous anastomoses [16], and vasoconstriction in this area is elicited by sympathetic nerve activation. An unpleasant taste, such as a bitter stimulus, can increase sympathetic nerve activity and consequently induce vasoconstriction, resulting in a decrease in nose skin blood flow. Conversely, there is no anatomical or physiological evidence to explain the observed eyelid skin blood flow responses.

Sour and salty stimuli themselves increased the skin blood flow in the eyelid and cheek, irrespective of the hedonic rating. The increase may be due to a reflex mechanism independent of hedonic information, unlike the case for the other taste stimuli. A sour stimulus induces a sweating response, known as gustatory flushing, while sweet and bitter stimuli do not [17]. This flushing is induced by the parasympathetic vasodilator reflex [9], [18] via the C-polymodal nociceptors [19] or transient receptor potential vanilloid-1 (TRPV1) receptors [20], [21]. TRPV1 receptors are activated by sour and salty tastes such as citric acid and NaCl, respectively, but not by sweet and bitter tastes such as sucrose and quinine, respectively [22]. Consequently, sour and salty stimuli could increase skin blood flow via the parasympathetic vasodilator reflex, independently of the hedonic rating.

The unique responses reported here were induced by changes in the peripheral circulation rather than in the central circulation, since no significant changes in either heart rate or MAP were detected. Some previous studies have suggested that basic taste stimuli increase the heart rate [4]–[6]; however, the subjects in these studies were not instructed to keep the face motionless. The formation of facial expressions such as angriness was shown to increase heart rate and finger skin temperature [23]. Since we confirmed the lack of electromyographic activity during all taste applications, the recorded facial skin blood flow responses were not related to movements of the facial muscles.

The functional importance of facial skin blood flow arises from the face easily conveying social signals. Facial expression may be associated with facial skin blood flow, since we often use phrases in daily conversational statements such as “you have a healthy complexion”, “you look pale”, and “you turned red with anger” (and their equivalents in other languages). In fact, fear and disgust decrease the skin temperature in the nose area of rhesus monkeys [13], [24], implying a change in skin blood flow. In addition, pain stimulation in a tooth decreases the skin blood flow and temperature in the nose area of humans [25]. Of the ANS-induced responses, the change in facial skin blood flow could be a useful key for communication. In this context, we should confirm in future research that peers are readily able to assess the rating of hedonic valence induced by tastes, using changes in skin blood flow as a key clue.

Differences between the study individuals did not affect the obtained results. The baselines appear to differ among Fig. 2a, b, and c, since data from different subjects are shown. We therefore calculated the relative value to estimate the response in skin blood flow whilst allowing for different baselines. Moreover, since the light output in laser speckle flowgraphy is automatically settled by the flowmeter, the baseline is arbitrarily determined. It is therefore meaningless to compare these baselines.

In summary, the findings of the present study demonstrate that sweet, umami, and bitter taste stimuli elicit characteristic facial skin blood flow responses, showing that facial skin blood flow mirrors a variety of complexions that reflect the hedonic valence. The responses observed in the eyelid and nose skin blood flows appear to represent signals of gustatory information. These findings imply that facial skin blood flow is a potentially objective method for assessing gustatory appreciation.

## Methods

Sixteen healthy adults (ten males and six females; age 26±5 years, mean±SD) participated in this study. The subjects were normotensive, not taking any medication, and did not report any gustatory disorder. The Ethics Committee of the Institution of Health Science, Kyushu University, Japan, approved the experimental protocol, and each subject provided written informed consent to participate prior to the commencement of the study.

Five taste solutions were used: sweet, sour, salty, umami, and bitter. Each taste was applied at three different concentrations (Table 2). Subjects were randomly given 1 ml of one of the five taste solutions at 37°C. They rated the taste as either pleasant or unpleasant on an 11-point hedonic scale (where 0 = no taste, +5 = the most pleasant taste, and −5 = the most unpleasant taste).

**Table 2:** The tested concentrations for each taste substance.

	Low	Medium	High
Sucrose	50 mM	300 mM	1000 mM
Citric acid	0.93 mM	56 mM	100 mM
NaCl	4.0 mM	160 mM	560 mM
MSG	3.0 mM	10 mM	60 mM
Quinine	0.01 mM	0.1 mM	0.5 mM

MSG: monosodium glutamate.

Subjects arrived at the laboratory after having abstained from exercise and alcohol ingestion for at least 1 day, and from eating, smoking, and consuming caffeine for at least 2 h, and they had not experienced sleep loss during the previous day. They were seated in a quiet room at an ambient temperature of 24°C, and participated in all of the trials. During the experiment, the subject rested his/her chin on a pedestal to stabilize the face, and was instructed not to move their facial muscles and to keep their eyes closed. After 20 min of resting, a “taste” solution was applied to the subject's oral cavity via a straw placed in the mouth. The order of the applied tastes was randomized, but the bitter solution was given last since the bitter taste persists for a long time. The concentrations for all types of taste were randomized, and the subject did not know the order in which the solutions were provided. The solution was kept in the mouth for 20 s before being spat out, and the mouth was rinsed with water at 37°C until the taste could no longer be perceived. The subject then reported his/her perceived hedonic valence.

Facial skin blood flow was measured for 4 s using laser speckle flowgraphy (LSFG-ANW-LL), before and 5 s after the onset of the taste stimulation. To assess skin blood flow in the forehead, eyelid, nose, cheek, upper lip, and lower lip, blood-flow images were constructed from the speckle images using commercially available software (LSFG Analyzer Cutaneous, Softcare, Fukuoka, Japan). Each pixel in the blood-flow image is regarded as a vector of an object's movement, and their sum within a given area thus corresponds to the relative value of the sum of that object's movements (i.e., blood flow). The target area in each subject's facial blood flow was kept constant.

The heart rate and MAP were measured. The beat-by-beat MAP was monitored with an automatic sphygmomanometer on the left middle finger (Finometer, Finapres Medical Systems, Amsterdam, The Netherlands). Furthermore, since the electromyographic activities of the zygomaticus major and corrugator supercilii muscles relate to movements in response to pleasant and unpleasant feelings [26], movements of these muscles on the right side were recorded.

The t-test revealed no gender differences in any of the study variables, and hence subsequent analyses were performed after combining data from male and female subjects. The hedonic scores related to water and each of the taste solutions were tested by one-way repeated-measures ANOVA and Bonferroni's post-hoc test. The facial skin blood-flow response was compared to a prestimulus baseline using a paired t-test. The relationship between hedonic score and facial skin blood flow was evaluated by Pearson's correlation. Heart rate, MAP, and integrated electromyography signals of the zygomaticus major and corrugator supercilii muscles were compared to the prestimulus baseline values using a paired t-test. The level of statistical significance was set at P<0.05. All statistical analyses were performed with SPSS (PASW statistics 18).
